# Acute toxicity of intraoperative radiotherapy and external beam-accelerated partial breast irradiation in elderly breast cancer patients

**DOI:** 10.1007/s10549-018-4712-3

**Published:** 2018-02-19

**Authors:** D. H. M. Jacobs, G. Speijer, A. L. Petoukhova, E. M. A. Roeloffzen, M. Straver, A. Marinelli, U. Fisscher, A. G. Zwanenburg, J. Merkus, C. A. M. Marijnen, M. E. Mast, P. C. M. Koper

**Affiliations:** 1 Department of Radiation Oncology, Leiden University Medical Center, Leiden, The Netherlands; 2 Department of Radiation Oncology, Haaglanden Medical Center, The Hague, The Netherlands; 3 Department of Radiation Oncology, Haga Hospital, The Hague, The Netherlands; 4 Department of Radiation Oncology, Isala, Zwolle, The Netherlands; 5 Department of Surgery, Haaglanden Medical Center, The Hague, The Netherlands; 6 Department of Surgery, Haga Hospital, The Hague, The Netherlands

**Keywords:** Elderly, Early-stage breast cancer, Accelerated partial breast irradiation, Intraoperative radiotherapy, Acute toxicity

## Abstract

**Background and purpose:**

We investigated the acute toxicity of accelerated partial breast irradiation using external beam (EB-APBI) or intraoperative radiotherapy (IORT) techniques in elderly breast cancer patients.

**Materials and methods:**

Women ≥ 60 years with unifocal breast tumors of ≤ 30 mm were eligible for this prospective multi-center cohort study. IORT was applied with electrons following lumpectomy (23.3 Gy). EB-APBI was delivered using 3D-CRT or IMRT in 10 daily fractions of 3.85 Gy within 6 weeks after surgery. Acute toxicity was scored using the CTCAE v3.0 at 3 months after treatment. Patient-reported symptoms were analyzed using visual analogue scales (VAS) for pain and fatigue (scale 0–10), and single items from the EORTC QLQ-C30 and Breast Cancer questionnaires.

**Results:**

In total, 267 (IORT) and 206 (EB-APBI) patients were available for toxicity analysis. More patients experienced ≥ grade 2 CTCAE acute toxicity in the IORT group (10.4% IORT and 4.9% EB-APBI; *p* = 0.03); grade 3 toxicity was low (3.3% IORT and 1.5% EB-APBI; ns); and no grade 4 toxicity occurred. EB-APBI patients experienced less fatigue direct postoperatively (EORTC *p* < 0.00, VAS *p* < 0.00). After 3 months only pain, according to the VAS scale, was significantly worse in the EB-APBI group (*p* < 0.00).

**Conclusion:**

Acute toxicity after IORT and EB-APBI treatment is acceptable.

## Introduction

Adjuvant whole breast irradiation (WBI) has shown to prevent local recurrence when administered as part of breast conserving therapy (BCT) for early-stage breast cancer patients [1–3]. However, WBI is inevitably associated with a significant treatment burden for the patient, given the number of fractions needed. Compliance to treatment is often suboptimal, especially for patients living further away from a radiation center [4, 5]. In addition, WBI can cause considerable damage to normal tissue resulting in fibrosis and skin toxicity [6]. Accelerated partial breast irradiation (APBI) encompasses the irradiation of solely the tumor bed, as recurrences most often occur in this area. This allows a higher dose per fraction and a shorter treatment duration while sparing healthy tissue. APBI has been studied elaborately over the past decades with several randomized trials proving similar tumor control after 5 years for APBI compared to WBI in selected patients [7–11]. GEC-ESTRO and ASTRO have provided guidelines for treatment selection for patients eligible for APBI inside and outside of clinical trials [12, 13].

APBI can be delivered with various techniques: intraoperative using either electrons or photons, externally or with brachytherapy. With IORT, by applying a single dose of radiotherapy to the tumor bed directly after lumpectomy, irradiation of the skin is avoided. Results regarding toxicity of electron IORT are scarce, but show less skin toxicity for IORT [10]. More data on toxicity are available for intraoperative radiotherapy using photons, but this technique differs substantially from electron IORT and this plausibly translates into different side effects [8]. For external beam APBI (EB-APBI), in which the skin still receives a significant dosage, the results regarding acute toxicity are variable [14–17].

More data on toxicity and safety of the different treatment options are important for patients eligible for APBI, as well as physicians considering to offer APBI as a treatment option for elderly patients. We set up a prospective cohort study of elderly (≥ 60 years) patients treated with either electron IORT or EB-APBI. In this analysis, we will report the acute (≤ 90 days after treatment) toxicity of these two treatment options.

## Materials and methods

### Patients

This prospective cohort study opened in 2011 in two participating centers initially: the Haaglanden Medical Center where patients were included to be treated with IORT, and the Haga Hospital where patients were included to be treated with EB-APBI. In 2015, a third center joined; the Isala in Zwolle accrued patients for the EB-APBI cohort. Inclusion was complete in November 2016. This was a non-randomized study; eligible patients received treatment according to center of inclusion. Inclusion criteria were identical for both cohorts.

Female patients aged 60 years or older, with invasive or in situ breast tumors of ≤ 30 mm (T1 and any hormonal receptor status or T2 and ER/PR positive and Her2neu negative), and clinical N0 status eligible for BCT and sentinel node procedure were eligible for this study. Exclusion criteria were multicentric or multifocal (> 2 cm from index lesion) tumors, extensive intraductal carcinoma or lymphovascular invasion, positive surgical margins, > pN1a after sentinel node procedure (or a positive sentinel node perioperatively in the case of IORT), neoadjuvant chemotherapy, previous malignancy in the past 5 years, or previous radiotherapy on the ipsilateral breast. These criteria correspond to patients classified as low or intermediate risk according to the 2010 GEC-ESTRO recommendations [13]. The study was approved by the medical ethical committee (10-042 METC ZuidwestHolland; NTR2931); patients accepted the study by signing informed consent.

### Surgery

Lumpectomy was performed either by palpation or localization procedure. A tumor-free margin was recommended and confirmed by specimen radiology. In the IORT group, perioperative visual inspection by a pathologist (IORT) was also performed. If the tumor-free resection margin was < 2 mm, an additional margin was removed directly by the surgeon. If both tumor margins and sentinel node were found to be negative by perioperative pathology, IORT was administered.

Gold (EB-APBI) or titanium (IORT) markers were applied for later identification of the lumpectomy cavity. Surgery was performed according to at least level 1 oncoplastic surgery principles [18]. In general, patients treated with IORT received prophylactic antibiotics preoperatively, in EB-APBI patients this was administered according to local protocol.

### Radiotherapy

IORT was administered directly after lumpectomy using an IORT dedicated mobile accelerator (Mobetron, INTRAOP, USA). A protection disc was placed under the lumpectomy site to protect the pectoral muscle, underlying ribs, and thoracic cavity. The electron tube diameter covered a total of 20 mm laterally of the lumpectomy cavity or clips, and ranged from 4 to 6.5 cm with a majority of 5 cm tubes used (41%). High-energy electron (6–12 MeV) beam radiotherapy was administered, delivering a total dose of 23.3 Gy (prescribed at the 100% isodose, according to ICRU 71) to the lumpectomy cavity. The electron energy was sufficient to apply 21 Gy at the 90% isodose for the full thickness of the glandular tissue.

EB-APBI was delivered within 6 weeks after surgery, in 10 daily fractions of 3.85 Gy using either Intensity Modulated Radiotherapy (*n* = 53) or 3D-Conformal Radiotherapy (*n* = 153). The CTV was defined as the region between the gold markers and seroma cavity added together, with an additional margin of 15 mm minus the lowest tumor-free resection margin. A CTV-PTV margin of 9 mm was used, excluding the skin.

Organs at risk were contoured, comprising both breasts, both lungs, the heart, the thoracic wall (ribs and musculature), and the skin. The dose was prescribed according to the ICRU 50 criteria. At least 98% of the PTV received ≥ 95% of the prescribed dose.

The treatment plans were carried out with 2–5 fields through use of ≥ 4 MV photons. To ensure quality of positioning, an online position verification procedure on the gold markers with Electronic Portal Images was used. Patients could be included only if ≤ 35% of the breast volume received 100% of the prescribed dose; if this was exceeded, patients were technically ineligible for EB-APBI.

### Adjuvant therapy

Patients received adjuvant treatment consisting of hormonal or chemotherapy according to the current Dutch breast cancer guidelines [19].

### Outcomes

Physician-reported toxicity was scored prospectively at a 3-month follow-up visit. The worst experienced toxicity up till 3 months after radiotherapy treatment was scored on a 5-point scale (none—slight—moderate—severe—surgery) regarding postoperative bleeding, hematoma, wound dehiscence, seroma, and infection. As this classification deviated from the prespecified CTCAE v3.0, we retrospectively scored toxicity according to the CTCAEv3.0 (Appendix 1) [20]. This was done by a blinded researcher with a medical background and checked by a blinded radiation oncologist for all patients with toxicities documented prospectively as “moderate” or worse.

Additionally patient-reported toxicity up till 3 months after treatment was analyzed. Patients were asked to fill out quality of life questionnaires at different time points: preoperatively, within 3 weeks after surgery, within 3 weeks after the last fraction of radiotherapy (for the EB-APBI cohort), and 3 months after either IORT or the last radiotherapy fraction. We analyzed six single questions of the EORTC QLQ30 and BR23 questionnaires to evaluate the following patient-reported symptoms: fatigue, pain, breast pain, swollen breast, oversensitive breast, and breast skin side effects. Scores of 1–2 (“not at all” and “a little”) were scored negative, and 3–4 (“quite a bit” and “very much”) were scored positive. Patient-reported pain and fatigue as reported on a Visual Analogue Scale (VAS) were also analyzed at time points after surgery, after EB-APBI and 3 months after radiotherapy treatment. Patients scored the amount of burden on a continuous scale from 0 till 10, where 0 was no symptoms at all and 10 the worst imaginable symptom burden.

Patients analyzed were all patients who had filled out the specified questions at the specified time point.

### Statistical analysis and study design

Data were collected in MSAcces^®^ and exported to SPSS 23^®^ (IBM SPSS statistics for Windows. Armonk, NY: IBM Corp.) for statistical analysis.

This non-randomized prospective cohort study was designed as a non-inferiority study with local breast recurrence at 5 years as the primary endpoint. Sample size calculation was done using one proportion non-inferiority power analysis. We deemed that local recurrence in this study could be 5.7% but should not exceed 10% at 5 years. This resulted in a sample size of 179 patients with invasive cancer per cohort (IORT and EB-APBI). This sample size provides sufficient power (80%) to detect the difference with a recurrence rate after BCT with WBI of 4% per 5 years (α = 0.05). Sample size was higher due to inclusion of pure DCIS.

Since the IORT cohort reached accrual before the EB-APBI cohort inclusion to the IORT cohort was continued until accrual to the EB-APBI cohort was complete to prevent lag-time bias inclusion. The primary endpoint will be reported in the future when follow-up time in both cohorts has matured. In this analysis we report acute toxicity, a prespecified secondary endpoint.

Patient characteristics in each cohort were described and compared using either the Mann–Whitney, independent *t* test or Chi-square test, depending on the type and distribution of the data.

Physician-reported toxicity at 3 months was compared using the Chi-square test. A two-tailed *p* value of ≤ 0.05 was deemed significant for both the patient characteristics and toxicity.

Patient-reported toxicity using single questions of the EORTC C30 and BR23 questionnaires was compared at the different time points between groups using the Chi-square test. The VAS scores were compared using the Mann–Whitney test, due to non-normal distribution of data. To correct for multiple testing for the patient-reported symptoms, a two-tailed *p* value ≤ 0.01 was used.

## Results

### Patients

**Fig. 1:**
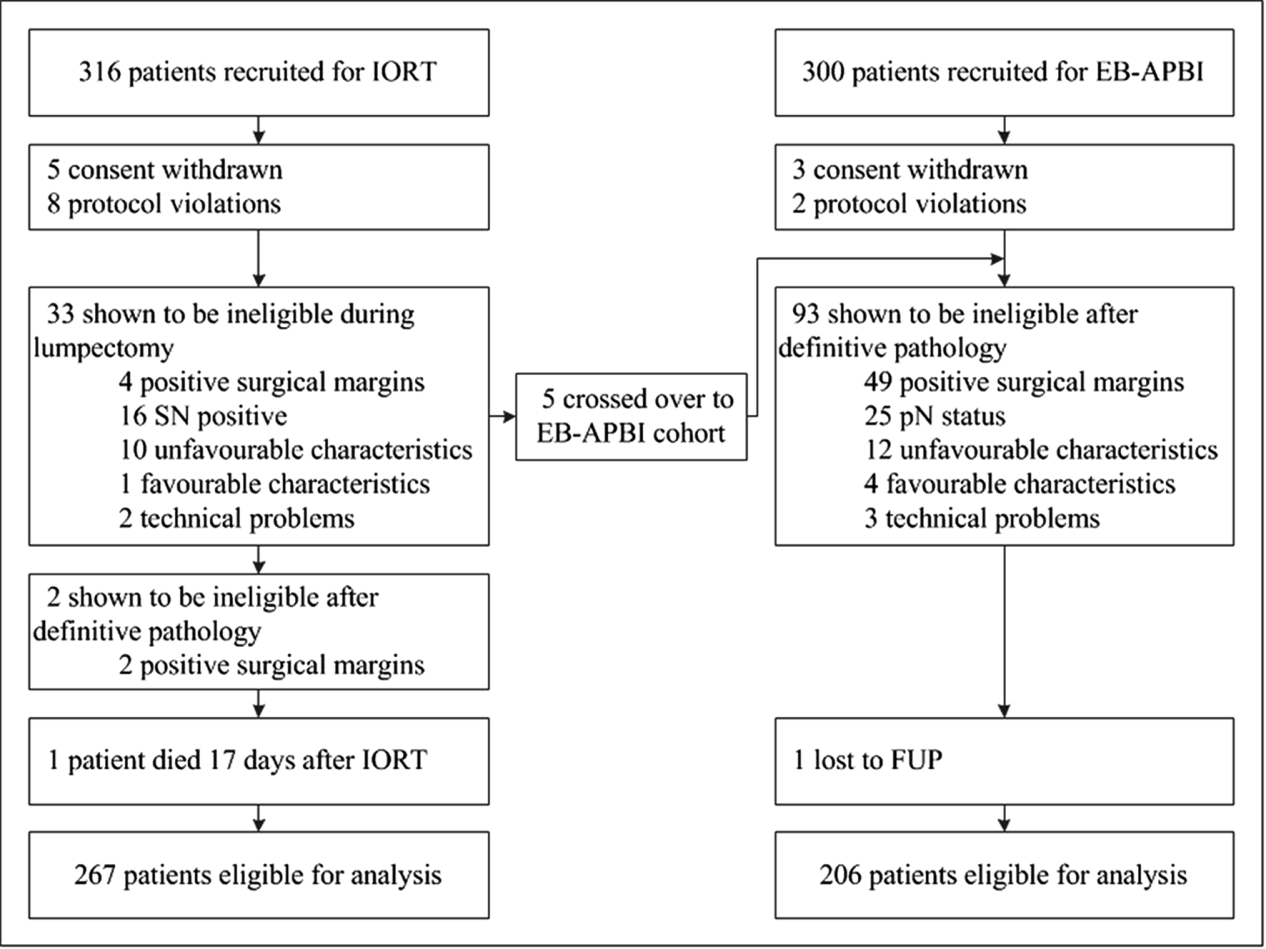
Patient inclusion flow diagram

Between January 2011 and November 2016, 316 patients entered the IORT treatment cohort and 300 patients the EB-APBI cohort. Figure 1 shows the patient inclusion flow chart for both cohorts.

Eventually 267 patients in the IORT cohort and 206 patients in the EB-ABPI cohort were eligible for analysis. In the IORT cohort 2 patients had bilateral breast cancer and both tumors were treated with IORT resulting in 269 treated tumors in 267 patients. Physician reported toxicity was scored separately for each breast tumor.

In Table 1, the patient characteristics of both groups are displayed. Median age was 68 and 67 years for the IORT and EB-ABPI group, respectively. Most tumors were estrogen receptor positive and low-grade (1–2). In 3/269, tumors were proven by preoperative biopsy but no residual tumor burden was found in the lumpectomy specimen (IORT). More patients in the IORT group had pN1mi or pN1a tumors.

**Table 1:** Patient characteristics

	IORT, *n* = 267^a^		EB-APBI, *n* = 206		*p* value
	*N*, median	%, range	*N, * median	%, range	
Age					
Years	68	59–90	67	59–86	0.683
pT stage					
pTis	18	7%	26	13%	0.068
pT1a	12	5%	14	7%	
pT1b	80	30%	50	24%	
pT1c	130	49%	89	43%	
pT2	26	10%	27	13%	
pN stage (invasive)					
pN0	227	92%	172	96%	0.045
pN1mi/pN1a	20	8%	6	3%	
unknown	1	0%	2	1%	
BR stage (invasive)					
Grade 1	81	33%	61	34%	0.075
Grade 2	109	44%	91	51%	
Grade 3	56	23%	25	14%	
unknown	2	1%	3	2%	
ER (invasive)					
Positive	232	94%	169	94%	0.712
PR (invasive)					
Positive	187	75%	138	78%	0.611
Her2neu (invasive)					
Positive	15	6%	10	6%	0.893
Systemic therapy					
No	157	59%	127	62%	0.538
HT	91	34%	60	29%	
CT ± HT	19	7%	17	8%	

^a^In the IORT group, 2 patients had bilateral breast cancer resulting in a total of 269 breast tumors treated with IORT. Three patients had pT0, biopsy-positive malignancies

Only 110/267 (41%) of IORT patients and 77/206 (37%) of EB-APBI patients received adjuvant hormonal therapy. The percentage of patients receiving chemotherapy alone was 1.9% and 2.5, 5.2 and 6% received a combination of systemic therapy comprising of CT, HT, and/or trastuzumab in the IORT and EB-APBI groups, respectively, according to Dutch breast cancer guidelines (19).

### Postoperative toxicity

Grade 2 or higher toxicity according to the CTCAE v3.0 was seen in 10.4% (28/269) of treated breasts in patients in the IORT group and in 4.9% (10/206) of patients in the EB-APBI group (*p* = 0.03). Grade 3 toxicity was present in 3.3% (9/269) of the treated breasts in patients in the IORT cohort and 1.5% (3/206) of patients in the EB-APBI cohort (*p* = 0.19). There were no grade 4 complications.

**Fig. 2:**
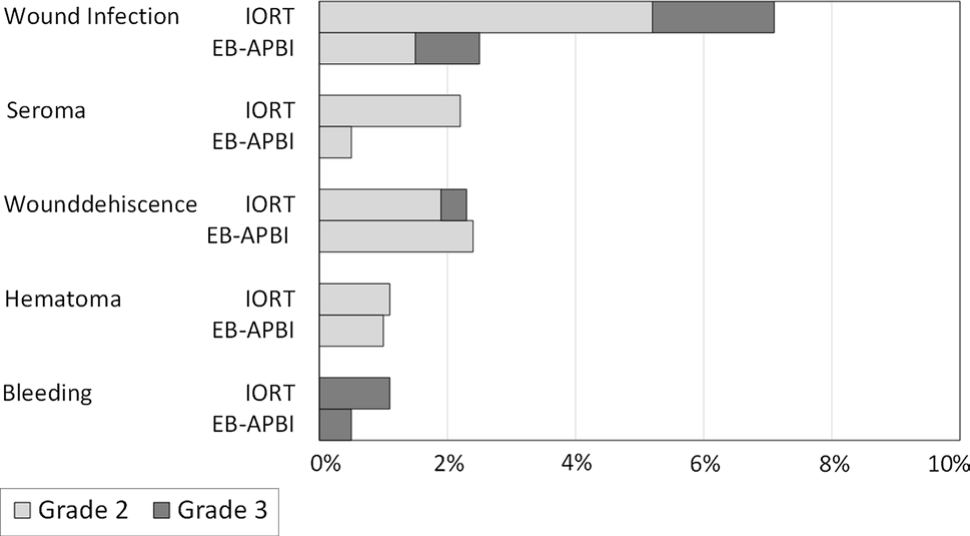
Toxicity according to CTCAE v3.0, in percentage of patients

Grade 2 and 3 toxicities per symptom are shown in Fig. 2. Different types of toxicity in one patient were scored separately. There were significantly more ≥ grade 2 wound infections in the IORT cohort (7.1% (19/269) IORT, 2.4% (5/206) EB-APBI, *p* = 0.02). Grade 3 wound infections occurred in 5/269 of treated breasts in IORT patients, one of which was an infection of a mammary prosthesis which had to be surgically removed. Two out of 206 patients in the EB-APBI cohort experienced grade 3 wound infection, one was treated surgically during EB-APBI treatment (radiotherapy was not interrupted and the wound closed 2.5 months later), the other was treated surgically and the infection resolved before start of radiotherapy.

### Patient-reported symptoms

**Fig. 3:**
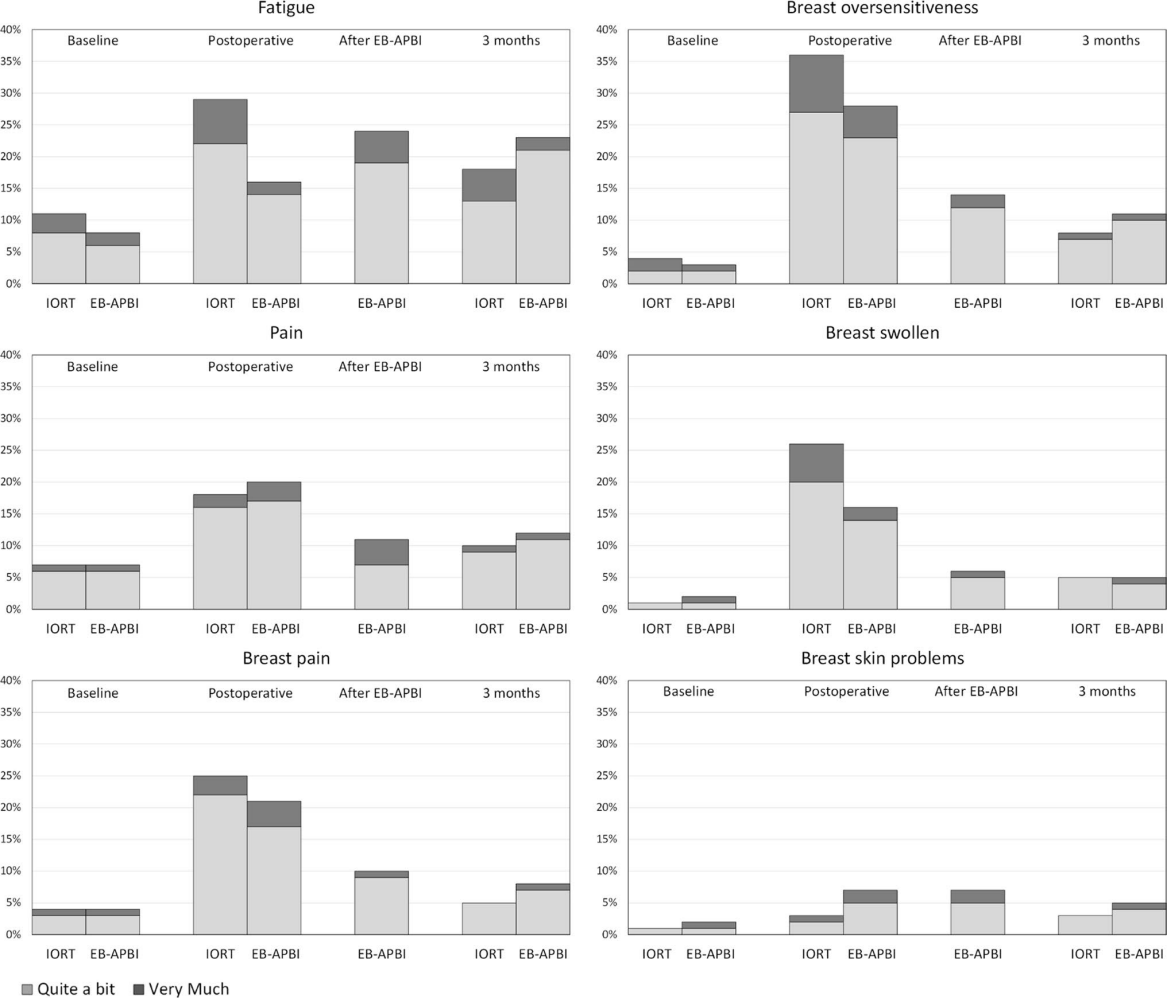
Patient-reported symptoms according to EORTC C30 and BR23 single items per time point. Percentage of patients reporting “quite a bit” or “very much” bother are shown

In Fig. 3, patient-reported symptoms for the six prespecified EORTC single items are shown. In Appendix 2, patient characteristics of responding patients per time point are shown.

At baseline, 3% more patients reported positive for fatigue in the IORT cohort (IORT 29/262, 11.1%; EB-APBI 15/176, 8.5%). All other symptoms at baseline differed ≤ 0.5% between cohorts.

**Fig. 4:**
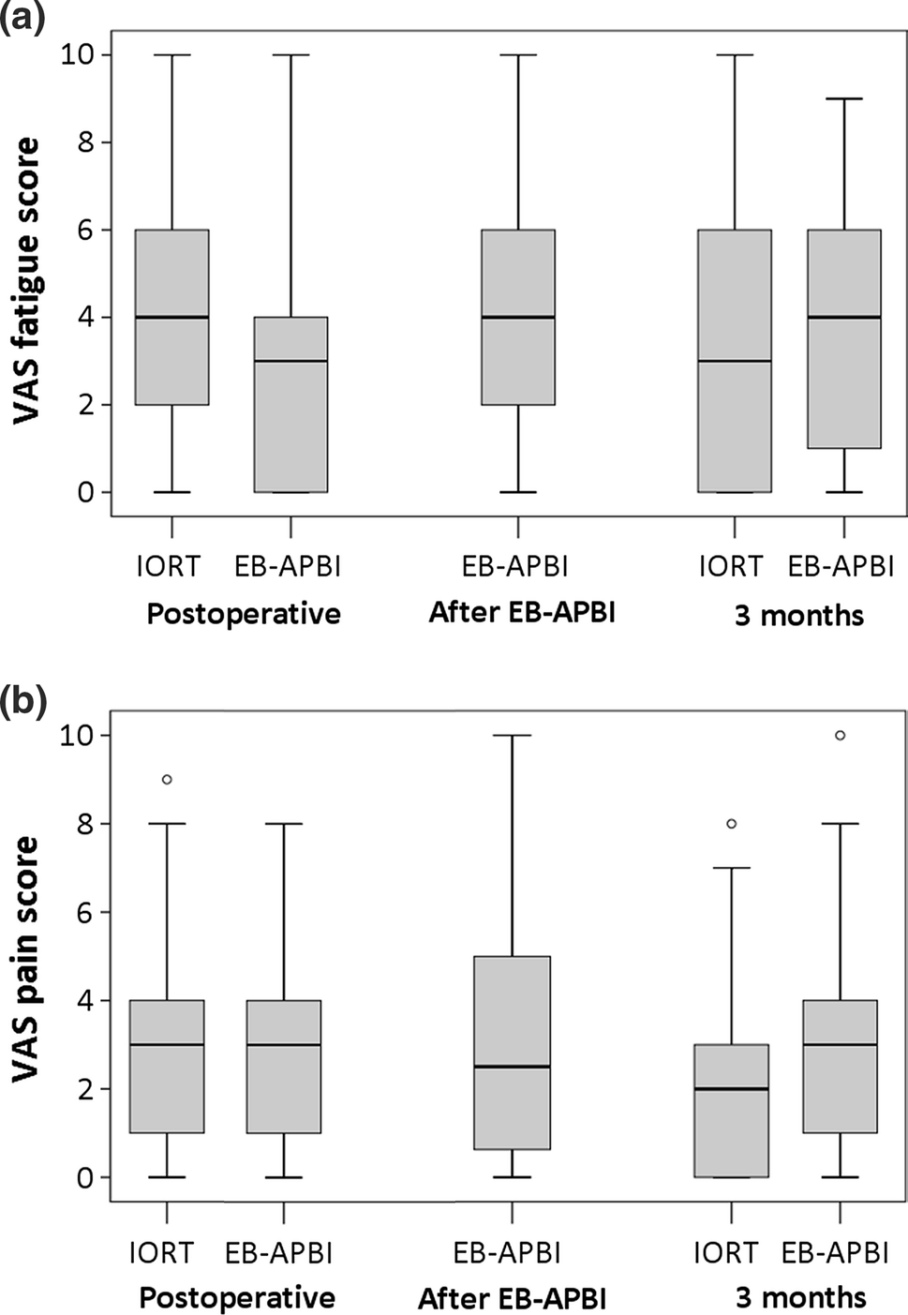
Patient-reported fatigue and pain according to VAS scores per time point. The boxplots represent the median and first and third quartiles. The whiskers represent minimum and maximum scores, and the circles represent outliers

Directly postoperatively, fatigue differed significantly between groups with 29.3% (65/222) of patients in the IORT group reporting positive for fatigue compared to 16.4% (22/134) of patients in the EB-APBI group (*p* < 0.00). When using the VAS scale, fatigue was also significantly worse in the IORT patients postoperatively (*p* < 0.00) (Fig. 4a).

The only patient-reported symptom that increased after EB-APBI compared to postoperatively was fatigue (from 16.4 to 23.9%). All other symptoms, including the VAS score for fatigue, either remained the same or decreased.

At 3 months, none of the EORTC patient-reported symptoms differed significantly between the two cohorts. Although not significant, patients treated with EB-APBI reported more Pain (EB-APBI 22/175 12.6%; IORT 24/245 9.8%; *p* = 0.37) and Breast pain (EB-APBI 16/175, 8%; IORT 14/245, 5.7%, *p* = 0.36) after 3 months according to the EORTC questionnaires. When asked on a VAS scale, EB-APBI patients reported significantly more pain at 3 months (*p* < 0.00) (Fig. 4b).

The general tendency of patient-reported symptoms was to increase postoperatively and recede towards baseline levels up till 3 months after treatment, with the exception of fatigue in the EB-APBI group (Figs. 3, 4).

## Discussion

The primary objective of this prospective analysis was to evaluate the acute (postoperative) toxicity of two different APBI treatment options for early-stage breast cancer patients ≥ 60 years. APBI has been extensively studied the past decades, but data on acute complications are scarce. The prospective evaluation of two different techniques of APBI focussing on an elderly patient group makes our study unique.

We have established that acute toxicity, postoperatively and up till 3 months after treatment, is acceptably low for both IORT and EB-APBI. Grade 3 toxicity was especially low, and there was no grade 4 toxicity. Directly after surgery breast symptoms, pain and fatigue are most prominent, after which they gradually decline. Overall, both APBI treatments are well tolerated by patients.

In current literature, results regarding toxicity of APBI treatments vary [14, 16, 21]. Different tools and alternating timings of measurement may explain some of this variation.

Probably even more important, however, is the fact that different techniques can be used for APBI, leading to different toxicity profiles. In addition, the influence of different surgical techniques and systemic therapy on toxicity, treatment compliance, and cosmetic outcome must be taken into account [22–24]. Therefore, extrapolating results from one type of APBI to another should be done with care.

By evaluating a prospective cohort of early breast cancer patients treated with two different types of APBI, we provide an overview of acute (postoperative) toxicity for the two techniques.

APBI has several benefits that especially facilitate treatment of elderly patients.

Firstly, APBI shortens treatment duration, an important factor for elderly patients as it influences not only patient compliance but also treatment choice [25]. Anticipated non-compliance to radiotherapy is often a reason to either overtreat, performing mastectomy to avoid irradiation, or undertreat, applying BCS without irradiation. Both treatments are suboptimal, as mastectomy is more mutilating and leads to more postoperative complications in elderly patients, especially in those with comorbidities [26, 27]. Although BCS without RT is considered safe in a selected group of elderly patients [28], it may lead to a higher local recurrence rate if applied to all elderly women [3, 29].

A common symptom related to treatment duration is fatigue. In our study, the difference in the course of fatigue between IORT and EB-APBI is noteworthy; IORT patients are significantly more fatigued after surgery, but EB-APBI patients experience increase in fatigue after radiotherapy which persists up till 3 months (Figs. 3, 4). Additionally, at 3 months, patients who received IORT reported less pain than patients treated with EB-APBI.

Secondly, the limited dose to normal tissue will lead to reduced toxicity, as compared to WBI. APBI techniques facilitate sparing of the skin, and in IORT no skin is irradiated at all. Accordingly, we observed very low skin toxicity, with 3% of patients in the IORT cohort and 5% of patients in the EB-ABPI cohort reporting skin problems after 3 months. In available literature, reported skin toxicity in EB-APBI treatment differs, suggesting technique and fractionation schedule influence skin toxicity [14–16, 21, 30]. Twice daily fractionation schedules are often used. However, the shortening of the recovery time between radiotherapy fractions might prevent adequate repair of normal tissue thus influencing skin toxicity and, in the long term, fibrosis [31]. Hence, once daily fractionation of EB-APBI seems preferable regarding skin toxicity.

Even though the irradiated volume using IORT is small, we saw more postoperative toxicity in the IORT group compared to EB-APBI. Perhaps the high dose of radiotherapy delivered during surgery damages tissue in such a way that it augments the chance of other complications such as seroma and wound infections. Moreover, with IORT a larger surgical bed is created and operation time is prolonged, resulting in a higher susceptibility for seroma and wound infections. Still, seroma was low in our IORT cohort and similar to seroma rates described for other types of IORT [8, 9, 32–35].Timing of APBI might also influence postoperative complications. In a study where EB-APBI was delivered preoperatively, 10% of patients developed persistent seroma, 11.4% of patients had a postoperative infection, and a total of 16% had postoperative complications [36].

One of the disadvantages of IORT may be that additional locoregional treatment such as mastectomy or axillary radiotherapy is needed after definitive pathology results become known. Examination of the specimen perioperatively resulted in cancelation of IORT in 10% of patients. Only 6/270 patients in the IORT group received additional locoregional radiotherapy treatment, and 2/270 required a mastectomy as a result of the definitive pathology outcome. Yet in 93/295 patients, EB-APBI was canceled due to unforeseen pathology results. Nevertheless, all patients that started EB-APBI completed the prescribed treatment (Fig. 1).

A strength of our study is that we included elderly patients, with a mean age of 68.5 years and 70% of patients aged ≥ 65 years. Elderly patients are underrepresented in randomized trials but are often suitable candidates for APBI due to favorable tumor features and conceivably they may benefit most from a less extensive treatment [25, 37].

A weakness of the current study is the treatment bias. Because of the nature of both treatments and the fact that IORT is only available in a limited number of centers, randomization between treatments was not achievable nor did we have the capacity to collect a control group receiving conventional WBI. Therefore, we chose to describe two cohorts receiving different types of ABPI using the same eligibility criteria, striving to create comparable groups. Given the fact that the two cohorts were collected in different centers, patient selection and surgical techniques may differ between centers. Therefore, firm conclusions regarding the differences between the two groups cannot be drawn.

The decision towards IORT, EB-ABPI, or even refraining from RT, will be influenced by patients’ preferences and tumor characteristics. Considering which aspects and outcomes of treatment are important for each individual patient deserves great emphasis during patient consultation. Not only the acute effect of treatment, but also the effect on fibrosis, long-term toxicity, cosmesis, and health-related quality of life must be further investigated to facilitate patients and physicians in well-informed shared decision-making.

## Conclusion

From literature, it can be concluded that although local control in elderly early breast cancer patients is acceptable with ABPI, the optimal treatment technique is yet to be defined. Despite the limitations of our study, we demonstrate that both IORT and EB-APBI are safe treatment modalities, with low acute toxicity and excellent compliance.

## Appendix 1

See Table 2.

**Table 2:** CTCAE v3.0

Adverse event	Grade				
	1	2	3	4	5
Wound infection	–	Localized, local intervention indicated	IV antibiotic, antifungal, or antiviral intervention indicated; interventional radiology or operative intervention indicated	Life-threatening consequences (e.g., septic shock, hypotension, acidosis, necrosis).	Death
Seroma	Asymptomatic	Symptomatic, medical intervention, or simple aspiration indicated	Symptomatic, interventional radiology, or operative intervention indicated	–	–
Wound dehiscence	Incisional separation of ≤ 25% of wound, no deeper than superficial fascia	Incisional separation > 25% of wound with local care, asymptomatic hernia	Symptomatic hernia without evidence of strangulation, facial disruption/dehiscence without evisceration, primary wound closure or revision by operative intervention indicated, hospitalization, or hyperbaric oxygen indicated	Symptomatic hernia with evidence of strangulation, fascial disruption with evisceration, major reconstruction flap, grafting, resection, or amputation indicated	Death
Hematoma	Minimal symptoms, invasive intervention not indicated	Minimally invasive evacuation or aspiration indicated	Transfusion, interventional radiology, or operative intervention indicated.	Life-threatening consequences, major urgent intervention indicated	Death
Hemorrhage with surgery	–	–	Requiring transfusion of 2 units non-autologous PRBC’s beyond protocol specification, postoperative interventional radiology, endoscopic, or operative intervention indicated	Life-threatening consequences	Death

## Appendix 2

See Table 3.

**Table 3:** Patient characteristics of EORTC and VAS responders at different time points

Variables	Preoperative			Postoperative			Post EB-APBI	3 months after treatment		
	IORT (*n* = 264)	EB-APBI (*n* = 181)	*p* value	IORT (*n* = 226)	EB-APBI (*n* = 134)	*p* value	EB-APBI (*n* = 173)	IORT (*n* = 245)	EB-APBI (*n* = 176)	*p* value
Age										
Median	68	67		68	67	0.369	67	68	67	0.369
pT stage										
pTis	7%	11%	0.066	8%	13%	**0.026**	13%	7%	13%	0.105
pT1a	5%	8%		4%	10%		7%	5%	7%	
pT1b	30%	24%		31%	27%		23%	30%	23%	
pT1c	49%	43%		48%	37%		43%	48%	43%	
pT2	10%	14%		9%	13%		14%	10%	14%	
pN stage (invasive)										
pN0	92%	95%	0.165	92%	94%	0.264	95%	92%	96%	0.409
pN1mi/pN1a	8%	4%		8%	4%		3%	7%	4%	
Systemic therapy										
Yes	42%	38%	0.546	42%	34%	0.288	38%	40%	39%	0.866

Significant differences between groups are marked in bold
